# Ischemic Stroke Secondary to Arterial Tunica Media Embolism Following Percutaneous Coronary Intervention: An Uncommon Etiology

**DOI:** 10.3390/diagnostics15131674

**Published:** 2025-06-30

**Authors:** Patricija Griškaitė, Neringa Jansevičiūtė, Givi Lengvenis, Kipras Mikelis, Mindaugas Zaikauskas, Marius Kurminas, Andrius Berūkštis, Algirdas Edvardas Tamošiūnas

**Affiliations:** 1 Vilnius University, Faculty of Medicine, Institute of Biomedical Sciences, Department of Radiology, Nuclear Medicine and Medical Physics, 03101 Vilnius, Lithuania; 2 Vilnius University, Faculty of Medicine, Institute of Clinical Medicine, Clinic of Cardiac and Vascular Diseases, 03101 Vilnius, Lithuania

**Keywords:** percutaneous coronary intervention complication, embolic stroke, tunica media embolus

## Abstract

Ischemic stroke following percutaneous coronary intervention (PCI) is a rare complication, with an overall incidence of 0.56%. Most embolic strokes result from the dislodgement of atherosclerotic plaques, thrombi formed on catheter surfaces, procedural maneuvers, or, less commonly, air or metallic emboli originating from fractured guidewires. We present a unique case of stroke following PCI due to a previously unreported mechanism—arterial tunica media embolization associated with arterial access. A 57-year-old female presented with chest pain at rest and with exertion, accompanied by episodes of anxiety and fluctuating blood pressure, for which coronary angiography was performed, revealing 90–99% stenosis of the left anterior descending artery and necessitating PCI. During the procedure, the patient developed an eye deviation, aphasia, and left-sided hemiparesis. Cerebral angiography identified a M2 segment occlusion of the right middle cerebral artery (MCA) and a subocclusion of the right anterior cerebral artery (ACA). Thrombectomy was performed, retrieving two white, tubular emboli resembling fragments of a vessel wall, histologically confirmed to be arterial tunica media. While PCI is associated with a low complication rate, its increasing frequency necessitates awareness of emerging complications. This case underscores a previously undocumented potential embolic complication arising from the performance of PCI.

**Figure 1:**
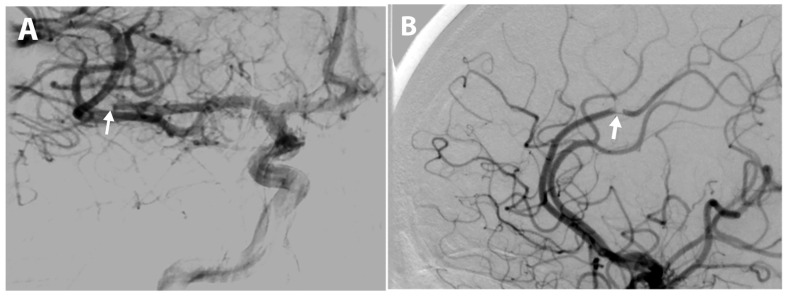
Cerebral angiography revealing (**A**) an occlusion in the M2 segment of the right middle cerebral artery and (**B**) an occlusion in the right anterior cerebral artery, both indicated by arrows. A 57-year-old female presented with chest pain at rest and with exertion, accompanied by episodes of anxiety and fluctuating blood pressure. Due to 90–99% stenosis of the left anterior descending artery revealed by coronary angiography, percutaneous coronary intervention was performed, during which vascular access was obtained by puncturing the right radial artery and a 6F radial sheath (Terumo Corp, Tokyo, Japan) was inserted. A 6F EBU 3.75 guiding catheter (Boston Scientific, Marlborough, MA, USA) was used. The stenosis of the RIA S7 segment was pre-dilated and stented with a 3.5 × 18 mm drug-eluting Ultimaster stent (Terumo Corp), followed by post-dilatation with a 3.75 mm NC emerge balloon (Boston Scientific). The final angiographic result was satisfactory. Nevertheless, near the end of the procedure, the patient abruptly exhibited an eye deviation, aphasia, and left hemiplegia, raising suspicion of an acute stroke. Due to a suspected occlusion of intracranial arteries, interventional radiologists were consulted and a cerebral angiography was performed (Figure 1).

**Figure 2:**
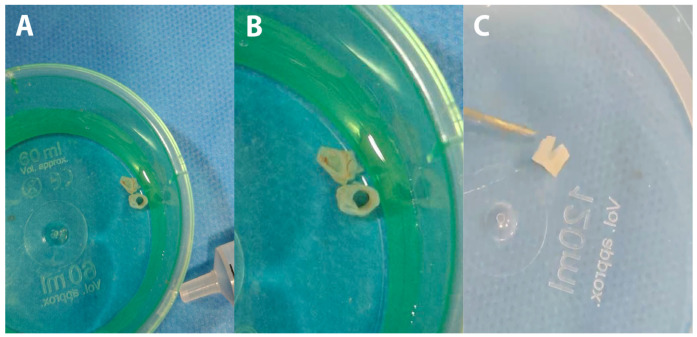
Using a FlowGate^2^ (Stryker Neurovascular, Fremont, CA, USA) balloon occlusion catheter and a 5F Sofia aspiration catheter (Terumo Neuro, Aliso Viejo, CA, USA) the MCA M2 thrombus was targeted. Advancement of the aspiration catheter over the thrombus was challenging, leading to an unsuccessful first pass with no retrieved embolic material. A second pass was then performed using a combined aspiration and stent retriever technique—a 5 × 40 mm pRESET stent retriever (Phenox, Bochum, Germany)—to “pinch” the embolus between the aspiration catheter and the stent retriever. The second pass was successful, the retrieved embolic material was white, tubular, and partially torn upon initial inspection, resembling fragments of a vessel wall (**A**–**C**). Using a similar technique with a smaller stent retriever device (Catch Mini, Balt, Montmorency, France) to address the ACA occlusion, an almost identical white, tubular embolus was also retrieved.

**Figure 3:**
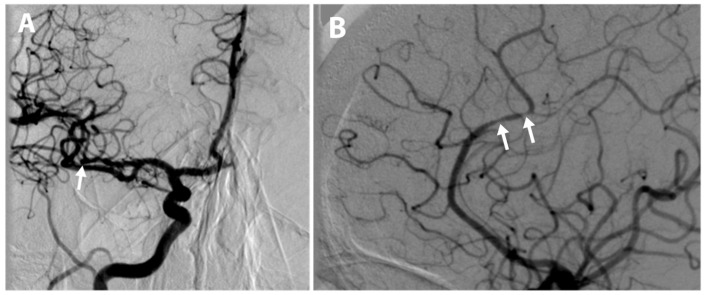
Post-thrombectomy cerebral angiography demonstrating restored blood flow in (**A**) the right middle cerebral artery, indicated by the white arrow, and (**B**) the anterior cerebral artery with vasospasm in the A3–A4 segments, indicated by the two white arrows. A follow-up head CT performed immediately after the procedure and 24 h later revealed no signs of acute ischemia or hemorrhage. The post-procedural National Institutes of Health Stroke Scale (NIHSS) score decreased from 10 immediately after the procedure to 5 the following day. On the follow-up day after the intervention, the patient’s neurological symptoms had resolved, with no speech or motor impairments observed, except for mild drowsiness. The patient’s condition upon discharge was stable, and further treatment was continued at the inpatient rehabilitation center.

**Figure 4:**
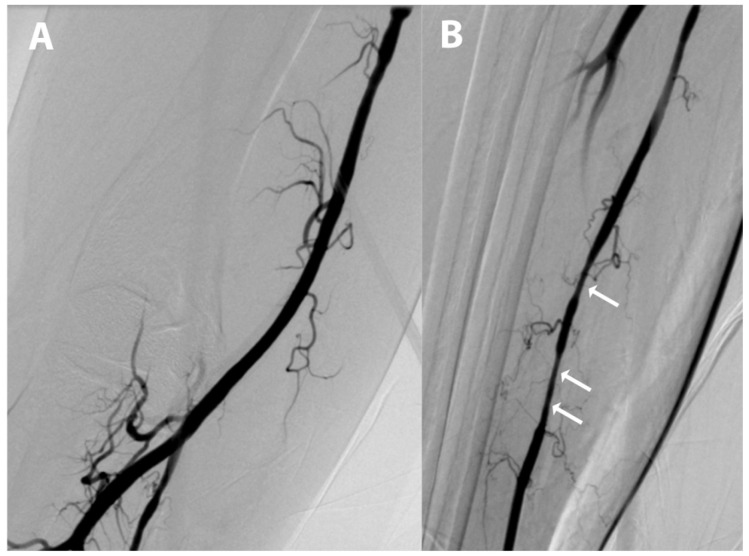
Suspecting a potential radial artery injury that led to dislodged vessel fragments, an angiogram of (**A**) the right brachial artery and (**B**) right forearm arteries was performed before the removal of the introducer sheath, revealing arterial spasm (indicated by the white arrows) but no apparent signs of dissection or extravasation. Radial access can be associated with intraprocedural complications, such as radial artery spasm, perforation, or dissection, as well as postprocedural complications including radial artery occlusion and pseudoaneurysm formation [1,2]. As reported in this case, the formation of a tunica media embolus may be a consequence of radial eversion endarterectomy, a rare access-site complication associated with radial artery spasm [1,2].

**Figure 5:**
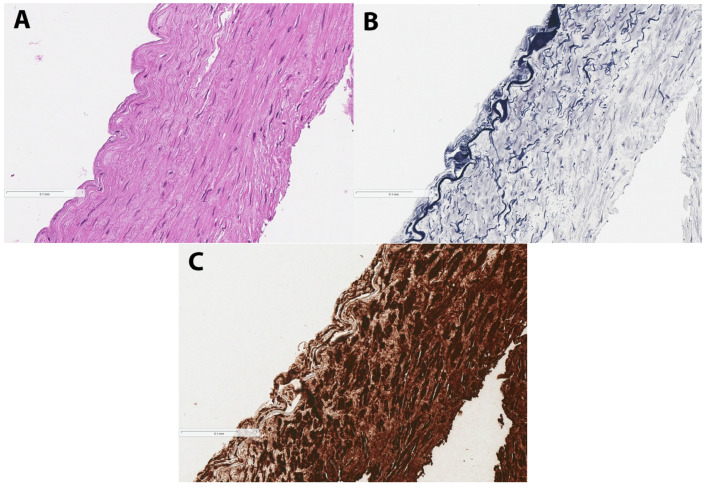
The retrieved emboli were submitted for histological examination, revealing (**A**) haematoxylin-eosin staining, identifying smooth muscle fibers, (**B**) van Gieson staining, highlighting elastic fibers and outlining the elastic membrane, and (**C**) immunohistochemical staining for alpha-smooth muscle actin (αSMA). A ready-to-use monoclonal anti-αSMA antibody, clone 1A4 (Agilent Technologies, St. Clara, CA, USA) was used. The staining revealed smooth muscle tissue. The magnification of histological images A, B, and C is 200×. These findings confirmed the structure of arterial tunica media. Given the histological evidence and the lack of any overt cardiac or coronary injury, we posit that the arterial tunica media emboli originated from the radial artery puncture site used for arterial access. While the transradial access demonstrates a lower incidence of complications compared with transfemoral intervention, it remains susceptible to intraprocedural complications such as radial artery spasm, perforation, or dissection, as well as postprocedural complications including radial artery occlusion and pseudoaneurysm formation [1,2,3,4]. Therefore, careful technique is essential during radial artery cannulation, sheath insertion, and the progression of wires or catheters, with early use of ultrasound and fluoroscopy advised to reduce procedural risk [1]. Despite the potential origin of the intima media embolus being the radial artery, follow-up angiography of the forearm did not reveal any extravasation, dissection, occlusion or other overt signs of artery injury (Figure 4). Moreover, no formation of a pseudoaneurysm was observed at the puncture site of the radial artery following the intervention.

## Data Availability

The original contributions presented in this study are included in the article. Further inquiries can be directed to the corresponding author.
